# Conservation and Divergence of the *CONSTANS-Like (COL)* Genes Related to Flowering and Circadian Rhythm in *Brassica napus*

**DOI:** 10.3389/fpls.2021.760379

**Published:** 2021-11-22

**Authors:** Yuxi Chen, Rijin Zhou, Qiong Hu, Wenliang Wei, Jia Liu

**Affiliations:** ^1^College of Agriculture, Yangtze University, Jingzhou, China; ^2^Oil Crops Research Institute of Chinese Academy of Agricultural Sciences/Key Laboratory for Biological Sciences and Genetic Improvement of Oil Crops, Ministry of Agriculture and Rural Affairs, Wuhan, China

**Keywords:** *Brassica napus*, *CONSTANS-LIKE (COL)* genes, genome-wide analysis, expression pattern, photoperiod

## Abstract

The *CONSTANS-LIKE (COL)* genes are important signaling component in the photoperiod pathway and flowering regulation pathway. However, people still know little about their role in *Brassica napus*. To achieve a better understanding of the members of the *BnaCOL* gene family, reveal their evolutionary relationship and related functions involved in photoperiod regulation, we systematically analyzed the *BnaCOL* family members in *B. napus* genome. A total of 33 *BnaCOL* genes distributed unevenly on 16 chromosomes were identified in *B. napus* and could be classified into three subfamilies. The same subfamilies have relatively conservative gene structures, three-dimensional protein structures and promoter motifs such as light-responsive *cis*-elements. The collinearity analysis detected 37 pairs of repetitive genes in *B. napus* genome. A 67.7% of the *BnaCOL* genes were lost after *B. napus* genome polyploidization. In addition, the *BnaCOL* genes showed different tissue-specific expression patterns. A 81.8% of the *BnaCOL* genes were mainly expressed in leaves, indicating that they may play a conservative role in leaves. Subsequently, we tested the circadian expression profiles of nine homologous genes that regulate flowering in *Arabidopsis*. Most *BnaCOL* genes exhibit several types of circadian rhythms, indicating that these *BnaCOL* genes are involved in the photoperiod pathway. As such, our research has laid the foundation for understanding the exact role of the *BnaCOL* family in the growth and development of rapeseed, especially in flowering.

## Introduction

Flowering is an important link in the process of plant reproduction (Fitter and Fitter, 2002). In *Arabidopsis thaliana*, the photoperiod pathway, vernalization pathway, autonomous pathway and gibberellin pathway constitute a complex genetic network that regulates flowering time (Roux et al., 2006). In *Arabidopsis*, *CONSTANS-like (CO/COL)* and *FLOWERING LOCUS T (FT)* are two important network regulation centers in the photoperiod induction pathway. *CO/COL* activates the transcription of FT to move the FT protein from the leaf phloem to the shoot apex meristem, thereby promoting plant flowering (Shim et al., 2017). In rice, the *Arabidopsis CO/COL* homologous gene *HEADING DATE 1* (*Hd1*) appears to be a bifunctional regulator. It induces FT homologous *HEADING DATE 3a* (*Hd3a*) gene expression to promote flowering under SD conditions while under LD conditions *Hd1* functions as an inhibitor of *Hd3a* transcription and flowering (Hayama et al., 2003).

*CO/COL* is an important network center of the photoperiod flowering pathway, integrating together various environmental and internal signals (Shim et al., 2017). Structurally, *CO/COL* genes contain two conserved domains: a C-terminal CCT domain (also termed CO, CO-like, TOC1) and an N-terminal zinc finger B-box domain (Robson et al., 2001). The B-box domains are found in many kinds of animal proteins, including some transcription factors, ribonucleoprotein and proto-oncogene products (Reddy et al., 1992; Borden, 1998), and it acts as a protein-protein interaction domain in several transcription factors in animals (Borden, 1998). The CCT domain has the function of nuclear localization similar to the yeast HEME ACTIVATOR PROTEIN2 (HAP2) protein and participates in DNA binding (Wenkel et al., 2006). In *Arabidopsis*, the 17 *AtCOL* genes can be classified into three subgroups according to the difference in structural domains (Robson et al., 2001): (i) *CO, COL1-COL5* form the first subgroup, and they all have two B-box domains and one CCT domain. (ii) The second subgroup consists of *COL9-COL15* members. Compared with members in other groups, they have a zinc finger domain in addition to a B-box domain and a CCT domain. (iii) The third subgroup includes *COL6-COL8* and *COL16* with one B-box and one CCT domain (Robson et al., 2001; Griffiths et al., 2003). However, there are exceptions in structural domains, such as *OsH* and *OsI* in rice and *HvCO9* in barley, which contain an intron and a CCT domain, but lack the B-box structure (Robson et al., 2001; Griffiths et al., 2003).

The *COL* gene family have been widely studied in angiosperms, such as monocotyledonous plants (rice, barley, maize, etc.) (Griffiths et al., 2003; Song et al., 2018) and dicotyledonous plants (*Arabidopsis*, soybean, cotton, tomato, etc.) (Robson et al., 2001; Wu et al., 2014; Cai et al., 2017; Yang et al., 2020). The *COL* genes function in all developmental stages of plants (Supplementary Table 1). In *A. thaliana*, *AtCOL1-AtCOL2* have less effect on flowering time, but overexpression of *AtCOL1* can shorten the cycle of circadian rhythm, and have a certain impact on the light input pathway (Ledger et al., 2001). Both *AtCOL3* and *AtCOL4* play an inhibitory role in flowering in both LD and SD (Datta et al., 2006; Steinbach, 2019). Besides, *AtCOL3* also promotes red light signal transmission, lateral root growth, bud branching and anthocyanin accumulation (Datta et al., 2006), while mutation of *AtCOL4* shows increased tolerance to ABA and salt stress (Min et al., 2015). Recently, *AtCOL3* and *AtCOL13* were found to be co-regulator of hypocotyl elongation under red light (Liu B. et al., 2021). Overexpression of *AtCOL5* will bloom early under SD conditions (Hassidim et al., 2009). *AtCOL7* not only affects the branching and shade response of *A. thaliana* (Wang et al., 2013), it is also a key factor linking light perception to auxin homeostasis (Zhang et al., 2014). And *AtCOL8* and *AtCOL9* transgenic plants flower late under LD conditions (Cheng and Wang, 2005; Takase et al., 2012). In rice, *OsCOL3*, *OsCOL4*, *OsCOL9, OsCOL10, OsCOL13, OsCOL15*, and *OsCOL16* act as flowering inhibitor to delay flowering time (Kim et al., 2008; Lee et al., 2010; Liu H. et al., 2016; Sheng et al., 2016; Tan et al., 2016, 2017; Wu et al., 2017, 2018), except *Ghd2*, which regulates leaf senescence and drought resistance (Liu J. et al., 2016). Furthermore, *CO/COL* genes are also found to regulate flowering time in potato (González-Schain and Suárez-López, 2008), sugar beets (Chia et al., 2008; Dally et al., 2018), soybean (Wu et al., 2014), sorghum (Yang et al., 2014), and bamboo (Xiao et al., 2018).

*Brassica napus* is an important and worldwide cultivated oil crop with strong adaptability, wide use and high economic value. Due to the great different cultivation in latitude, longitude and climate, different ecological types of *B. napus* varieties are needed. Hence, it is also a good plant resource to research flowering pattern and photoperiod rhythm like spring ecotype, winter ecotype and semi-winter ecotype. And the *CO/COL* genes are important transcription element in photoperiod and flowering regulation pathway. At present, the functions of *CO/COL* gene family members have been comprehensively studied in the model plant *Arabidopsis thaliana*, but little is known about *CO/COL* genes preservation and functional differentiation in *B. napus* after polyploidy events. In this study, we have identified 33 *BnaCOL* gene family members and performed bioinformatics analysis on their physical and chemical properties, evolutionary relationships, chromosome location, gene structure, three-dimensional protein structures, *cis*-acting elements of the promoter, GO annotation enrichment analysis and gene duplication. We also studied the expression patterns of *BnaCOL* gene family in different tissues and their response to SD or LD light treatment. This study would provide important clues for the functional study of the *COL* gene family in the Cruciferae plants, and lay a foundation for further exploration of its functional and molecular mechanisms.

## Materials and Methods

### Identification of *CO-like* Transcription Factor Family in Rapeseed

The genome sequences, protein sequences and gene annotation files of rapeseed were downloaded from the website (BnPIR)^1^ (Song et al., 2020). The Markov model of the two domains of CO-like CCT (PF06203) and zinc finger B-box (PF00643) was downloaded from Pfam database^2^. Using these two Markov models to preliminarily screen the protein sequences of rapeseed on the HMMER software, and the cut-off *E*-value were set to1e-4, respectively. Subsequently, all candidate proteins were submitted to three online websites, i.e., SMART^3^, NCBI CDD^4^ and PFAM (see text footnote 2) to screen out candidate COL proteins with both CCT and B-box conserved domains. The identified *COL* candidate genes were submitted to the ExPASy website^5^ for prediction analysis of protein molecular weight (MW) and isoelectric point (pI). And subcellular localization is predicted by WoLF PSORT^6^.

### Chromosome Location and Phylogenetic Analysis

The chromosome location data of *BnaCOLs* comes from the BnPIR website (see text footnote 1). And then the MapChart software was used to analyze the distribution of the identified *BnaCOLs* on rapeseed chromosomes. The results were refined with Adobe Illustrator software.

According to the reported literature (Hu et al., 2018), the protein sequences of *COL* family members of *Arabidopsis*, *B. oleracea*, *B. rapa*, *Capsella rubella*, *Oryza sativa*, *Raphanus sativus*, and *Zea mays* were downloaded and Clustal W was used to analyze the COL protein sequences of these plants. In addition, the sequence alignment results were submitted to MEGA 7.0 software, and the neighbor joining method (NJ) was used to construct the evolutionary tree (Saitou and Nei, 1987; Kumar et al., 2016).

### Gene Structure and Protein Conservative Domain Analysis

The exon and intron structures of *COL* genes in rapeseed were analyzed by Gene Structure Display Server 2.0^7^ (Hu et al., 2015). The BnaCOL protein sequences were submitted to the MEME software to analyze the conserved domain of genes. Setting the maximum number of motifs to 10, the maximum number of motif amino acids to 20 and the minimum width to 6 and other settings to default. Finally, TBtools software was used to visualize the conserved motifs of BnaCOL proteins.

### Multi Sequence Alignment and Three-Dimensional Structure Prediction of Protein

We submitted 33 protein sequences of BnaCOL to DNAMAN7.0 software for multiple sequence comparison. Subsequently, we used the online website Phyre2^8^ to predict the three-dimensional structure of the protein.

### Collinearity Analysis Within *Brassica napus* and Among Different Species

The analysis of intra-species collinearity of *BnaCOL* genes in *B. napus* was performed with McScanX software and the relationship was plotted with Circos software. In addition, the collinearity analysis was plotted with Python version of McScanX software.

### *Cis*-Acting Element and Functional Annotation Analysis

The 1,500 bp upstream sequences of *BnaCOL* genes were obtained from *B. napus* Whole Genome Information Resource Website (see text footnote 1). The online website Plant CARE^9^ was used to extract homeopathic a components, and then using the online website DSGS to visualize.

To shed light on the function of the *BnaCOL* genes, we used eggNOG database^10^ for the gene ontology (GO) annotation analysis. Subsequently, the GO annotation data was processed in TBtools.

### Tissue-Specific Expression Pattern of *BnaCOL* Genes

At the online website BnTIR: *Brassica napus* transcriptome information resource^11^ (Liu D. et al., 2021), we downloaded RNA-seq data of different tissues including roots, cotyledons, leafs, sepals, petals, pollen, buds, siliques, and seeds. The data were submitted to the online tool^12^ to draw the expression heat map.

### Plant Materials and Treatment Methods

The seeds of Zhongshuang 11 were grown in a growth chamber with a temperature of 25°C/18°C, light for 16 h/darkness for 8 h and humidity of 80%. When the seedlings were at the five leaf stage, two different photoperiod treatments were applied: long daylight (LD, 16 h light/8 h dark) and short daylight (SD, 8 h light/16 h dark). We collected the third leaf of these seedlings at 0, 4, 8, 12, 16, 20, and 24 h after photoperiod treatment. Besides, we set up three biological replicates with samples collected. The collected leaves were immediately frozen in liquid nitrogen and then stored in −80°C refrigerator.

### RNA Extraction and RT-PCR Analysis

Total RNA was extracted from leaves treated with different photoperiodic treatments using polysaccharide polyphenol total RNA extraction kit (Tiangen Biochemical Technology Co., Ltd: DP201101X). The quantity and quality of RNA was determined by an ultramicroscopic spectrophotometer (Thermo Fisher, NanoDrop One). We used a reverse transcription kit to synthesize cDNA and diluted 100 times with ddH_2_O as templates for subsequent RT-qPCR experiments. Based on the coding sequences of *BnaCOL* genes, specific primers were designed using online website qPCR Primer Database^13^. All *BnaCOL* genes primers were listed in the Supplementary Table 1. SYBR^®^ Premix Ex Taq^TM^ (TaKaRa) was used for the real-time quantitative experiment. In this experiment, three biological replicates were collected and the samples without photoperiod treatment were used as controls. The gene relative expression analysis refers to the 2^−ΔΔ*Ct*^ method.

## Results

### Identification of *CO-Like* Transcription Factor Family in Rapeseed

We have identified 33 *COL* genes in *B. napus* and named them *BnaCOL1-BnaCOL33* (Table 1). Subsequently, the physical and chemical properties of all members were analyzed and predicted. The lengths of the proteins encoded by *BnaCOL* genes varied from 289 to 414 amino acids, the MW ranged from 31.51 to 46.5 kDa, and the PI ranged from 5.08 to 8.10. The other information about all BnaCOL proteins were list in Table 1, including subcellular location prediction and coding sequence length.

**TABLE 1:** The position and molecular information of *COL* gene family in *B. napus*.

Gene name	Gene ID	Chromosomes position	CDS (bp)	Protein			Subcellular localization prediction
				Length (bp)	MW (kDa)	pI	
*BnaCOL1*	BnaA01G0416100ZS	36950508 – 36951440 +	932	310	34.32	5.45	Nuclear
*BnaCOL2*	BnaA02G0061400ZS	3266686 – 3267639 −	953	317	35.45	6.83	Chloroplast
*BnaCOL3*	BnaA02G0116400ZS	6119846 – 6121295 −	1,027	342	37.59	5.49	Chloroplast
*BnaCOL4*	BnaA02G0174500ZS	10523483 – 10524893 −	1,195	398	45.13	5.39	Mitochondria
*BnaCOL5*	BnaA02G0360300ZS	31881593 – 31883093 −	1,050	350	38.77	5.59	Nuclear
*BnaCOL6*	BnaA04G0165100ZS	17420919 – 17421857 +	868	289	31.51	7.99	Chloroplast
*BnaCOL7*	BnaA05G0182600ZS	12667807 – 12668866 +	943	314	37.01	5.95	Nuclear
*BnaCOL8*	BnaA06G0333200ZS	41587921 – 41589471 +	1,051	350	37.87	5.69	Cytoplasm
*BnaCOL9*	BnaA06G0365400ZS	43546693 – 43548134 +	1,073	358	39.4	5.85	Nuclear
*BnaCOL10*	BnaA07G0096900ZS	14681867 – 14683344 +	924	308	34.03	5.8	Nuclear
*BnaCOL11*	BnaA07G0106200ZS	15211667 – 15213016 −	1,225	408	45.99	5.51	Chloroplast
*BnaCOL12*	BnaA07G0275200ZS	25743942 – 25745385 +	1,207	402	45.62	5.91	Nuclear
*BnaCOL13*	BnaA09G0066400ZS	3989150 – 3991166 −	1,039	346	37.39	5.77	Cytoplasm
*BnaCOL14*	BnaA09G0437200ZS	49558950 – 49560928 −	1,243	414	46.5	5.08	Nuclear
*BnaCOL15*	BnaA10G0134500ZS	17803925 – 17805427 +	1,185	353	38.78	6.21	Chloroplast
*BnaCOL16*	BnaA10G0206100ZS	22123527 – 22124540 +	1,013	337	37.94	6.57	Nuclear
*BnaCOL17*	BnaA10G0206200ZS	22132539 – 22133811 +	1,090	363	41.25	6.73	Nuclear
*BnaCOL18*	BnaC01G0499200ZS	56776905 – 56777870 −	965	321	35.75	5.58	Nuclear
*BnaCOL19*	BnaC02G0071200ZS	4363404 – 4364369 −	965	321	35.88	7.89	Chloroplast
*BnaCOL20*	BnaC02T0142900ZS	10195224 – 10196603 −	1,027	342	37.48	5.63	Chloroplast
*BnaCOL21*	BnaC02G0484200ZS	58992489 – 58993974 −	1,041	347	38.38	5.5	Nuclear
*BnaCOL22*	BnaC03G0630900ZS	60651107 – 60652647 +	1,168	389	43.39	6.49	Nuclear
*BnaCOL23*	BnaC04G0461800ZS	58868888 – 58869910 +	940	313	33.61	7.52	Chloroplast
*BnaCOL24*	BnaC05G0226300ZS	17178698 – 17180607 +	1,243	414	46.36	5.12	Chloroplast
*BnaCOL25*	BnaC05G0309900ZS	28672694 – 28673760 +	943	314	36.99	6.2	Nuclear
*BnaCOL26*	BnaC06G0312000ZS	41664896 – 41666349 +	1,216	405	46.01	6.02	Chloroplast
*BnaCOL27*	BnaC07G0158900ZS	28546979 – 28548324 −	1,222	407	45.8	5.77	Nuclear
*BnaCOL28*	BnaC07G0159900ZS	28645926 – 28647275 −	1,225	408	45.83	5.45	Chloroplast
*BnaCOL29*	BnaC07G0327300ZS	46665480 – 46667263 −	1,076	359	39.62	5.85	Nuclear
*BnaCOL30*	BnaC07G0361000ZS	48935280 – 48936860 −	1,051	350	37.74	5.46	Cytoplasm
*BnaCOL31*	BnaC09G0055900ZS	3580725 – 3582245 −	1,033	344	37.34	6.27	Cytoplasm
*BnaCOL32*	BnaC09G0505400ZS	60998624 – 60999625 +	1,001	333	37.53	6.51	Nuclear
*BnaCOL33*	BnaC09G0505500ZS	61005355 – 61006629 +	1,099	366	41.71	8.10	Nuclear

*MW, molecular weight; pI, isoelectric point.*

### Chromosome Location and Phylogenetic Analysis

**FIGURE 1:**
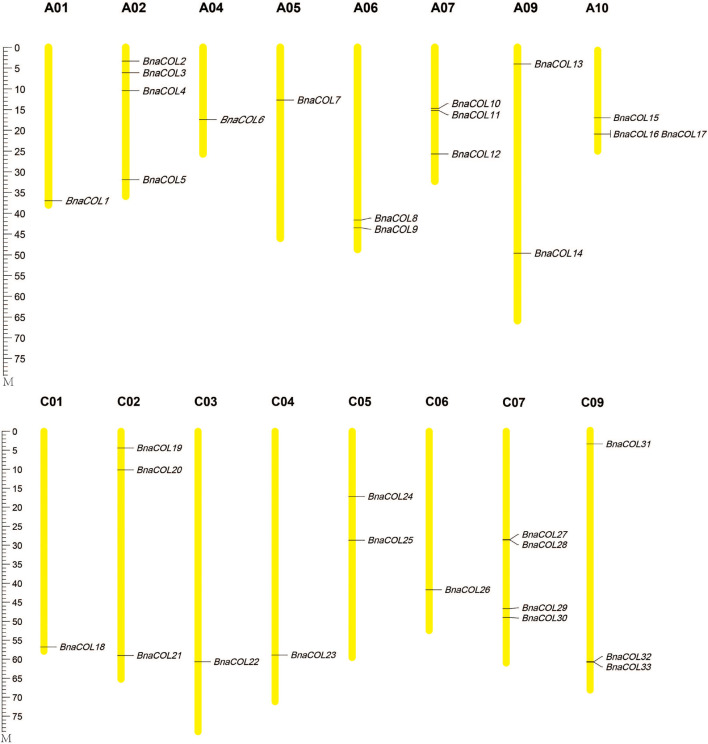
Chromosomal localization of *BnaCOL* genes in *B. napus.* The chromosomes with different sizes are represented by the yellow vertical bars with different lengths. The locations of genes are shown from **top** to **bottom**.

Furthermore, the 33 *BnaCOL* genes were mapped on chromosomes (Figure 1). These 33 *COL* genes were distributed unevenly across 16 chromosomes in rapeseed genome and no gene distributed on chromosome A03, A08, and C08. There was only one gene located on chromosome A01, A04, A05, C01, C03, C04, and C06; and two genes on chromosome A06, A09, C05; three genes on chromosome A07, A10, C02, and C09; four genes on chromosome A02 and C07.

**FIGURE 2:**
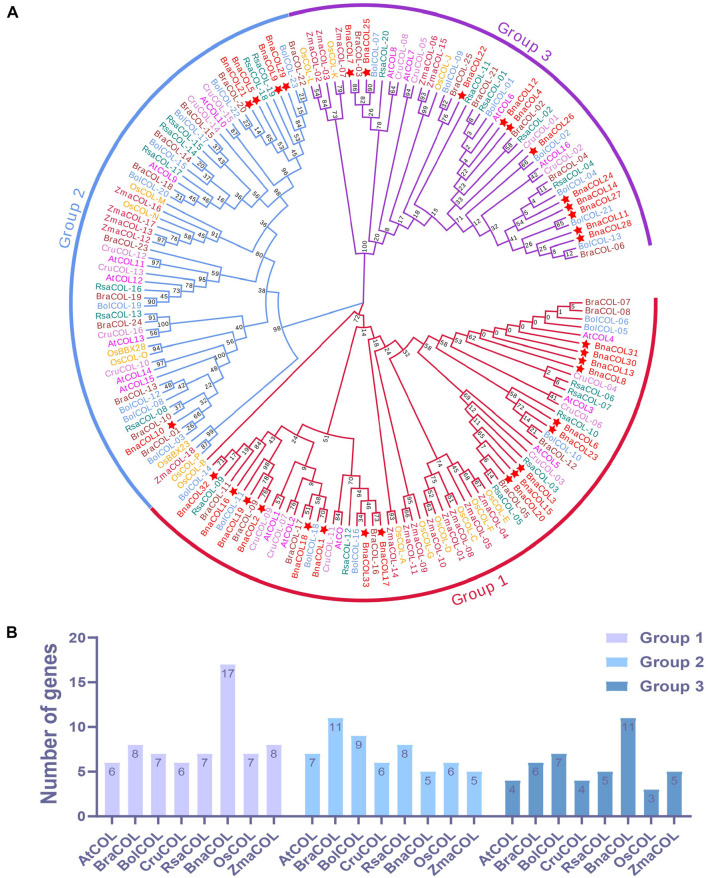
Phylogenetic analysis and number statistics of *COL* homologs in different species. **(A)** Phylogenetic tree using 137 *COL* proteins from 7 species, including *rice, maize, Arabidopsis*, *B. napus*, *B*. *oleracea*, *B. rapa*, and *radish*. The clades of group 1, 2, and 3 are marked in red, blue, and purple, respectively. Among them, *BnaCOLs* are represented by red five-pointed stars. The abbreviations represent the species as follows: Os, *Oryza sativa*; Zma, *Zea mays*; At, *Arabidopsis thaliana*; Bna, *Brassica napus*; Bol, *Brassica oleracea*; Bra, *Brassica rapa*; Cru, *Capsella rubella*, and Rsa, *Raphanus sativus*. **(B)** Statistics of the number of *COL* genes in each group. The ordinate is the number of genes and the abscissa is the *COL* genes of each species.

To gain a better understanding of the evolutionary relationship between the *COL* genes of different species, we constructed a phylogenetic tree using 137 *COL* proteins from seven species, including *Arabidopsis*, *B. oleracea*, *B. rapa, B. nigra*, rice, radish, maize (Protein sequences are shown in Supplementary Table 2). As shown in Figure 2A, these *COL* genes were classified into three groups. The first group consisting of seven species contained the most *COL* members, while the third group had the least numbers of *COL* genes. For the *BnaCOL* genes, there were 17, 5, and 11 members clustered in to the group 1, 2, and 3, respectively (marked with asterisks in Figure 2A). The *BnaCOL* members which were closely grouped may come from a common origin and have similar functions.

Subsequently, we counted the number of *COL* genes of each species in each group (Figure 2B). Based on the number of *COL* genes in *A. thaliana*, only about one copy of the *COL* genes were retained in each group of species. It should be pointed out that the *COL* genes of *B. napus* in Group 1 and Group 3 retain about 3 homologous copies.

### Protein Conservative Domain and Gene Structure Analysis

**FIGURE 3:**
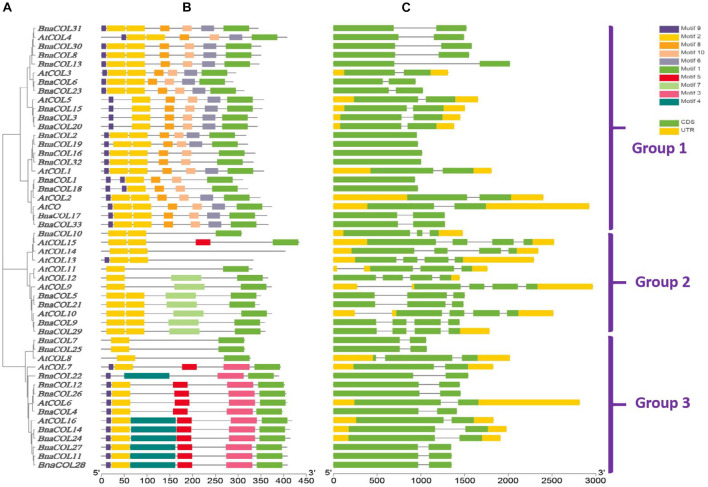
Comparative analysis for conserved domain and gene structure between *AtCOL* genes and *BnaCOL* genes. **(A)** The phylogenetic analysis of 17 *AtCOL* genes and 33 *BnaCOL* genes divided them into three groups: 1, 2, and 3. **(B)** The conserved domains of AtCOL and BnaCOL proteins. Schematic representation of the 10 conserved motifs in BnaCOL proteins, different colored boxes represent different motifs. **(C)** The gene structure of *AtCOL* and *BnaCOL* genes. The green box represents exons, the yellow box represents UTR and the black line represents introns.

To investigate the structural diversity of *BnaCOL* genes, we constructed a phylogenetic tree using 17 AtCOL protein sequences from *A. thaliana* and 33 BnaCOL protein sequences from *B. napus.* All of the *COL* genes were classified into three groups: 1, 2, and 3 (Figure 3A). And then their protein conserved domains and gene structure were further analyzed.

The protein conserved domain analysis revealed a total of 10 different conservative motifs (Figure 3B). In general, all members contained motif 1 (CCT domain) and motif 2 (B-box domain), indicating that CCT domain and B-box domain are highly conserved in *BnaCOL* genes. Besides, similar conserved motifs were found in members of the same group. For example, all members of group 1 contained six motifs: motif 9, motif 2, motif 8, motif 10, motif 6, and motif 1 and the distribution and length of these motifs were consistent. Furthermore, most members of group 2 contained three motifs, among which motif 7 only existed in group 2, while the most *BnaCOLs* in group 3 contain six motifs, among which motif 5, motif 3 and motif 4 are unique to members of group 3. However, there were slight differences in the number and distribution of *COL* motifs in different groups. In group 2, four members, i.e., *BnaCOL10, AtCOL13, AtCOL14*, and *AtCOL15* contained conserved motifs different from other members. In group 3, *BnaCOL7, BnaCOL25*, and *AtCOL8* had three fewer motifs than other members.

The gene structure analysis showed that the *BnaCOL* genes in the same group usually had similar exons and introns (Figure 3C). Both group 1 and 3 contained two exons and one intron. But there was a little difference in the distribution and quantity of exons and introns in group 2.

### Multi Sequence Alignment and Three-Dimensional Structure Prediction of Protein

To elucidate the structural characteristics of BnaCOL proteins, we carried out multiple sequence alignment (Supplementary Figure 1) and three-dimensional structure prediction analysis (Supplementary Figure 2). On the basis of these results we concluded that these proteins have highly conserved CCT and B-box 1 domains, but the sequence of B-box 2 is slightly different.

Then we further predicted the three-dimensional structure of the B-box domain, the results are in good consistent with previous research results (Li et al., 2020). We divided the BnaCOL proteins into three groups according to their genetic relationship. Most of the B-box structure is similar. It is worth noting that group C only contains the predicted B-box 1 domain but not the B-box 2 domain.

### Collinearity Analysis Within *Brassica napus* and Among Different Species

**FIGURE 4:**
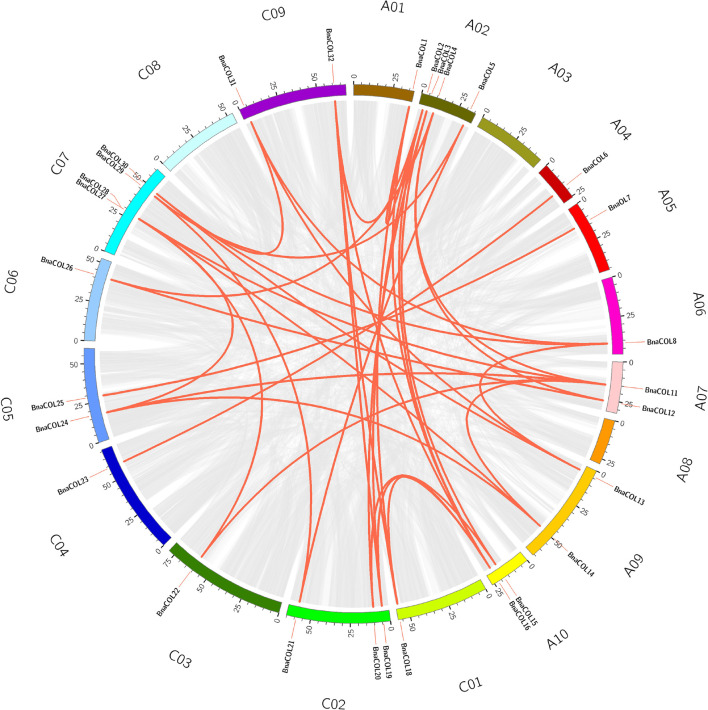
Collinearity analysis of the AC subgenome of *COL* genes in *B. napus.* Among them, the gray line represents the replication event of all genes in *B. napus* and the red line represents tandem repeat events within the *BnaCOL* genes.

Genome wide replication analysis is of great importance for the origin, evolution and genome expansion of species. We hence analyzed the *COL* gene family replication events in *B. napus* to understand the causes of *BnaCOL* genes replication events. The results showed that 37 pairs of large fragment repeat genes were detected (Figure 4) and fragment repeats were found on 17 chromosomes except for A03 and C08. These results indicated that large fragment replication may be a major driving force for the amplification and evolution of *COL* genes in *B. napus* genome.

**FIGURE 5:**
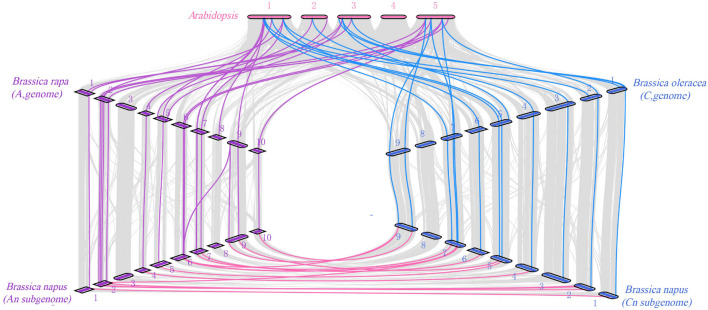
Syntenic relationship of *COL* genes in *B. napus* and three ancestral plant species. The figure shows the collinearity between *Arabidopsis* (*A. thaliana*), *Brassica rape* (*B. rapa*), *Brassica oleracea* (*B. oleracea*), and *Brassica napus* (*B. napus*).

In order to trace the evolutionary process of the *COL* gene family in *Brassica*, we analyzed the homologous relationship among *Arabidopsis*, *B. napus* (A and C subgenomes), *B. rapa* (A genome), and *B. oleracea* (C genome) (Figure 5). The collinearity analysis showed that there were a large number of orthologous *COL* genes in *Arabidopsis*, *B. rapa, B. oleracea*, and *B. napus.* There were 19 pairs of genes in *Arabidopsis* and *B. rapa* that showed collinearity, 13 *B. rapa COL* genes had homologous genes in *Arabidopsis*, among which 6 were multi-copy genes and 7 were single-copy genes. In addition, *B. rapa* lacked homologous genes of *AtCOL2, AtCOL7, AtCOL11*, and *AtCOL14*, which indicated that gene loss happened in *B. rapa* during evolution. Moreover, 17 pairs of genes in *Arabidopsis* and *B. oleracea* showed collinearity, and 12 *B. oleracea COL* genes had homologous genes in *Arabidopsis* while homologous genes of *AtCOL5, AtCOL7, AtCOL11, AtCOL13*, and *AtCOL14* were not found. The A and C subgenomes of *B. napus* were mainly collinear with the corresponding diploid *B. rapa* and *B. oleracea*. The A genome of *B. napus* and *B. rapa* had 16 homologous gene pairs, while 14 homologous gene pairs were found between the C genome of *B. napus* and *B. oleracea*. For the evolution of *COL* gene family, although gene loss occurs, the vast majority of *COL* genes remain intact in *B. napus*.

### *Cis*-Acting Element and Functional Annotation Analysis

**FIGURE 6:**
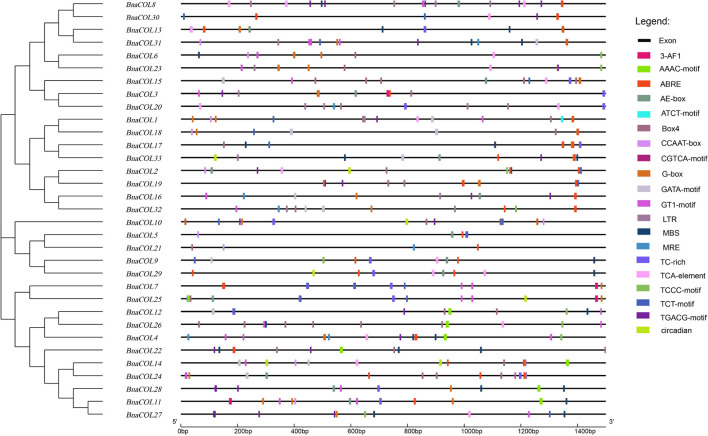
*Cis*-acting element analysis of *COL* gene family in *B. napus.* The boxes with different colors on the black line represent different *cis* elements.

The *cis*-acting element is the binding site of transcriptional regulators and regulates gene transcription. In order to investigate the potential function of the *BnaCOL* genes, we analyzed the *cis*-acting elements of the upstream sequence of the *BnaCOL* promoters at 1,500 bp and excluded the elements with unknown function and the general transcriptional regulatory elements (Table 2 and Figure 6). These *cis*-acting elements can be broadly classified into four categories, which involved in light response, hormonal response, growth regulation, and abiotic-stress response. Among them, the components involved in the light reaction include G-box, GATA-motif, Box4, TCT-motif, ATCT-motif, Circadian, AAAC-motif, AE-box, TCCC-motif, GT1-motif, 3-AF1 binding site and MRE. Hormone response elements include TCA-element, TGACG-motif and CGTCA-motif, ABRE. In addition, several stress response elements such as TC-rich repeats, LTR, MBS were observed. These results showed that most of the *BnaCOL* genes had photoresponsive elements indicating that *BnaCOL* genes may played a critical role in the regulation of photoreactivity.

**TABLE 2:** The *cis*-elements have been identified in more than three *BnaCOL* genes.

Site name	Sequence	Function of the *cis*-elements
G-Box	CACGTG	*cis*-acting regulatory element involved in light responsiveness
GATA-motif	AAGATAAGATT	part of a light responsive element
TCA-element	CCATCTTTTT	*cis*-acting element involved in salicylic acid responsiveness
Box 4	ATTAAT	part of a conserved DNA module involved in light responsiveness
TCT-motif	TCTTAC	part of a light responsive element
ATCT-motif	AATCTAATCC	part of a conserved DNA module involved in light responsiveness
circadian	CAAAGATATC	*cis*-acting regulatory element involved in circadian control
TC-rich repeats	ATTCTCTAAC	*cis*-acting element involved in defense and stress responsiveness
LTR	CCGAAA	*cis*-acting element involved in low-temperature responsiveness
AAAC-motif	CAATCAAAACCT	light responsive element
AE-box	AGAAACAA	part of a module for light response
TCCC-motif	TCTCCCT	part of a light responsive element
GT1-motif	GGTTAAT	light responsive element
TGACG-motif	TGACG	*cis*-acting regulatory element involved in the MeJA-responsiveness
CCAAT-box	CAACGG	MYBHv1 binding site
CGTCA-motif	CGTCA	*cis*-acting regulatory element involved in the MeJA-responsiveness
ABRE	ACGTG	*cis*-acting element involved in the abscisic acid responsiveness
3-AF1 binding site	TAAGAGAGGAA	light responsive element
MRE	AACCTAA	MYB binding site involved in light responsiveness
MBS	CAACTG	MYB binding site involved in drought-inducibility

Taking the above observations in account, we performed GO annotation and enrichment analysis of *BnaCOL* genes to gain a better understanding of their function. The analysis results mainly included three aspects: biological process (BP), molecular function (MF) and cellular component (CC) (Supplementary Table 4 and Supplementary Figure 3). In the biological process (BP), most genes were annotated in light signal response and transmission, photoperiod response, flowering regulation, circadian rhythm, etc. This is consistent with the observation from the *cis*-acting element. In the molecular function (MF), a total of 11 highly enriched items were detected, including the combination of DNA, protein and organic compounds and transcription regulator activity. Likewise, in the cellular component (CC), most gene annotations were located on the nucleus and organelles. This indicates a good consistency between the prediction of subcellular location and GO enrichment analysis.

### Tissue-Specific Expression Pattern of *BnaCOL* Genes

**FIGURE 7:**
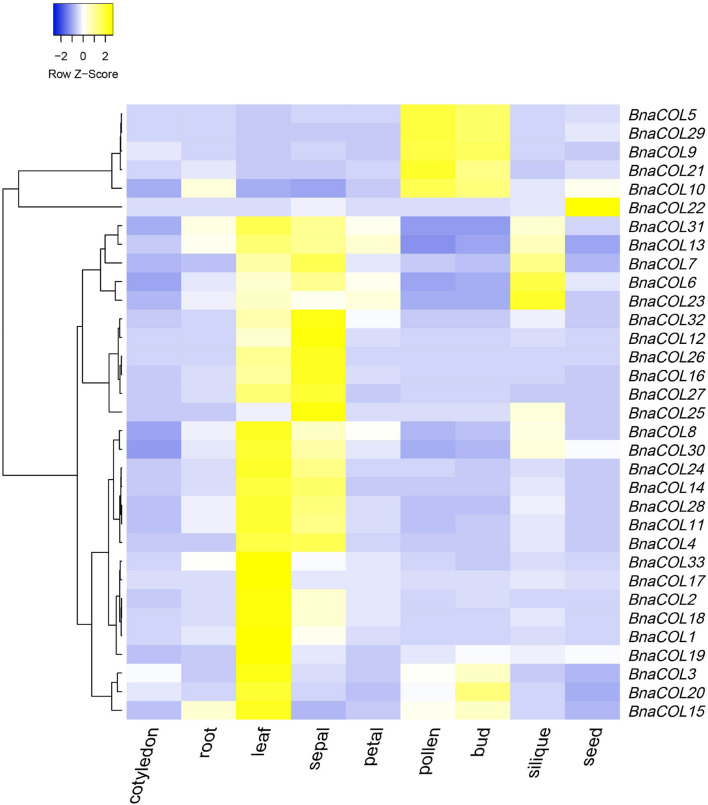
The heat map shows the expression profile of *BnaCOL* genes in different tissues. The transcriptome data origins from the online website (BnTIR: *Brassica napus* transcriptome information resource).

To further study the expression patterns of the *BnaCOL* genes in different tissues of rapeseed, we used the online website (BnTIR: *Brassica napus* transcriptome information resource) to download the rapeseed genome-wide transcription data of different tissues. As show in Figure 7 and Supplementary Table 5, all *BnaCOL* genes showed different expression characteristics in various tissues, which indicated that *BnaCOL* genes were usually not tissue-specific genes. A 81.8% of *BnaCOL* genes showed high expression levels in leaves and sepals, while *BnaCOL5, BnaCOL9, BnaCOL10, BnaCOL21*, and *BnaCOL29* were abundantly expressed in pollen and flower buds. Nevertheless, *BnaCOL6, BnaCOL7, BnaCOL13, BnaCOL23*, and *BnaCOL31* were not only highly expressed in leaves and sepals, but also highly detected in siliques. *BnaCOL6* and *BnaCOL23* expressed the highest levels in siliques. In particular, the expression of *BnaCOL22* was lower in other tissues, but highest in seeds. On the basis of these results we concluded that the *BnaCOL* gene family were critical for all stages of the development of rapeseed individuals and some members with similar expression characteristics may perform similar functions.

### Diurnal Rhythm of Expression of *BnaCOL* Genes

**FIGURE 8:**
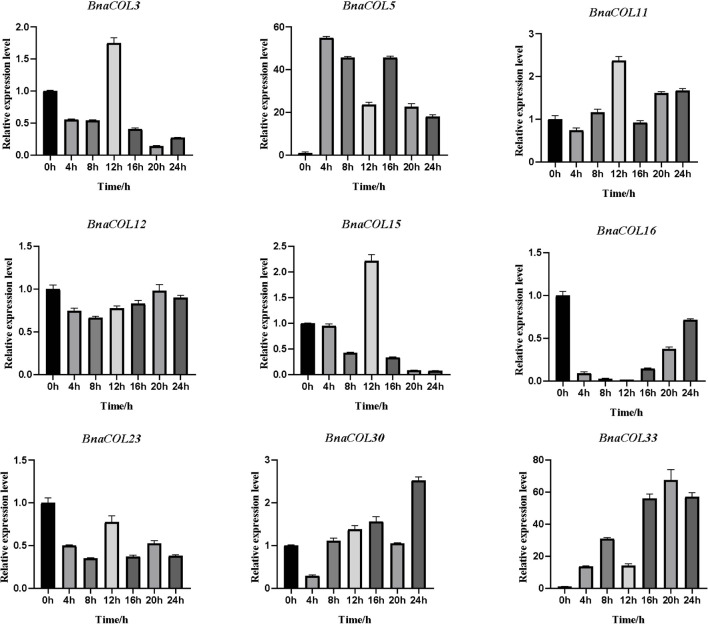
The expression pattern of *BnaCOL* genes under LD conditions. Under LD (16 h dark/8 h), the third true leaf was collected every 4 h. The error column represents the standard deviation of the three biological replicates.

**FIGURE 9:**
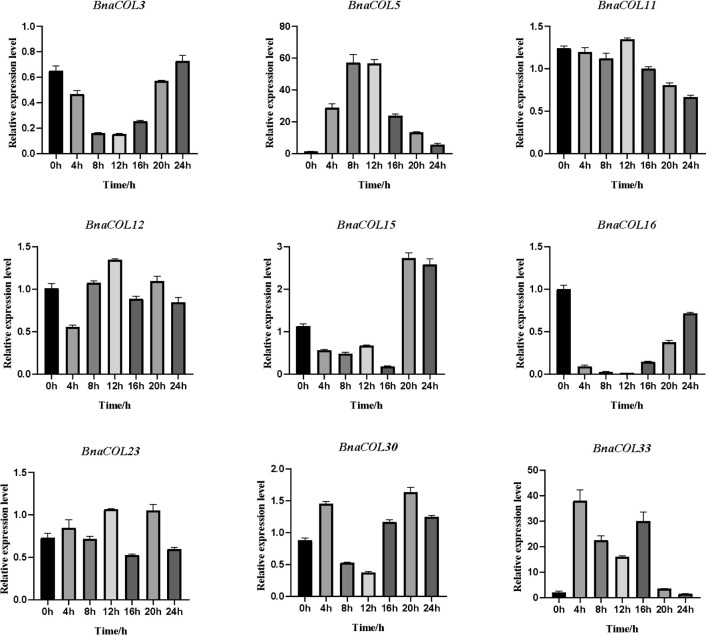
The expression pattern of *BnaCOL* genes under SD conditions. Under SD (8 h light/16 h dark), the third true leaf was collected every 4 h. The error column represents the standard deviation of the three biological replicates.

Previously, we analyzed the *cis*-acting elements in the upstream sequence of the *BnaCOL* promoters and found that most *BnaCOL* genes have light-responsive elements, indicating that they may involve in photoperiod regulation. To further identify the possible function, we selected nine homologous genes that regulate flowering in *Arabidopsis* (*BnaCOL3, BnaCOL5, BnaCOL11, BnaCOL12, BnaCOL15, BnaCOL16, BnaCOL23, BnaCOL30, BnaCOL33*) and tested the circadian expression profile of these nine genes within 24 h (Figures 8, 9).

The circadian expression pattern of *BnaCOL* genes under LD illumination showed four types: (i) The expression of *BnaCOL3, BnaCOL11, BnaCOL15*, and *BnaCOL23* increased slowly during illumination, peaked at 12 h and then rapidly decreased to a lower level. (ii) While *BnaCOL30* and *BnaCOL33* had similar expression patterns, their expression levels presented a stepwise increase pattern. Whether it is day or night, they reach their peak in 20 h or 24 h. (iii) On the contrary, *BnaCOL5* was up-regulated before 4 h, then gradually declined. (iv) In particular, the expression level of *BnaCOL16* showed light inhibition and dark induction. The expression reached its peak at 0 h and decreased rapidly to a lower level under light conditions, then slowly increased to dark. However, *BnaCOL12* had no significant difference in expression level under LD illumination.

The circadian expression pattern of *BnaCOL* genes under SD illumination showed three types: (i) The expression level of *BnaCOL3, BnaCOL16*, and *BnaCOL30* gradually decreased during the light period, decreased to a minimum at 8 h or 12 h and then slowly rised, showing U-shaped curve. (ii) However, the expression levels of *BnaCOL5, BnaCOL11, BnaCOL12*, and *BnaCOL23* increased slowly from 0 h, reached a peak at 12 h, whereafter gradually dropped down. (iii) It was interesting to find that the expression level of *BnaCOL15* was lower during the light period and reached the highest at 20 and 24 h under dark conditions, indicating that the dark treatment can activate the expression of *BnaCOL15*.

In general, most *BnaCOL* genes exhibited different diurnal expression patterns, indicating that these *COL* genes were involved in the photoperiod pathway. However, the expression patterns of *BnaCOL12* and *BnaCOL16* were similar under LD or SD conditions, indicating that these two genes may not be affected by photoperiod.

## Discussion

In this study, we conducted a biological analysis of the *COL* gene family of *B. napus* and related species, including their chromosomal location, phylogenetic analysis, gene structure, protein conserved domain analysis and three-dimensional structure prediction of protein. The results showed that 33 *BnaCOL* family members were unevenly distributed on 16 chromosomes of rapeseed. Based on phylogenetic analysis, these members were divided into three groups. And most of the genes in the same subgroup had similar gene structure, protein conserved domains and three-dimensional protein structure, which reflects the conservation of the *BnaCOL* gene family.

### The Retention and Deletion of *CONSTANS-LIKE* Genes in *Brassica* During Evolution

Most angiosperms (including monocots and dicots) have experienced whole-genome duplications (WGDs). In the dicotyledon *A. thaliana*, it has experienced three WGDS events: two ancient tetraploid events (β, α) and one ancient hexaploid (γ) event (Bowers et al., 2003; Tang et al., 2008). And previous studies have shown that the ancestral genomes of Brassica species (similar in structure to *A. thaliana*) have undergone genome-wide triploid replication events, indicating that they evolved from a common hexaploid ancestor (Lysak et al., 2005; Town et al., 2006). *B. napus* (A and C genomes) is the amphidiploid species formed by a cross between *B. rapa* (A genome) and *B. oleracea* (C genome). As such, the rape genome contains six times the ancestral genome (Parkin et al., 2002). During the evolution of *Brassica*, it remains question to be explored whether the retention and deletion of flowering and photoperiod genes like *COL* accompanying with the evolution of *Brassica* genome.

In the process of polyploidization, copy number variation is an important way of evolution. Taking the number of *COL* genes in *Arabidopsis* as a reference, the number of *COL* genes in *B. oleracea*, *B. rapa, B. nigra*, radish, rice and maize in each group only retained roughly one copy. We speculate that the *COL* genes have basically undergone recombination and fusion during the evolution process. It should be noted that the retention rate of *BnaCOL* genes in the three groups were quite different. In the first group, 47.2% of the *BnaCOL* genes were retained. In the second group, only 11.9% of the *BnaCOL* genes remained. The third group, the *BnaCOL* genes retention rate was 45.8%. On the basis of our findings, it can be concluded that the *AtCOL* genes of the first and third groups had about three orthologous genes in *B. napus*, while the *AtCOL* genes of the second group only retained one copy in *B. napus*, indicating that the *COL* gene family shrank during the diversification of various species. These missing *COL* genes may be redundant genes, gradually being replaced by other genes with similar functions.

To further explore the evolutionary process of the *COL* gene family in *Brassica*, we analyzed the replication events of the *COL* gene family in *B. napus.* The results revealed a total of 37 pairs of large-segment repetitive genes existed. We also analyzed the homology relationships among *Arabidopsis*, *B. napus*, *B. rapa*, and *B. oleracea*. The results of collinearity analysis showed that there were a large number of homologous *COL* genes in *B. napus*, *Arabidopsis*, *B. rapa*, and *B. oleracea*. However, *B. rapa* lacks homologous genes of *AtCOL2*, *AtCOL7, AtCOL11*, and *AtCOL14*, while *B. oleracea* lacks homologous genes of *AtCOL5, AtCOL7, AtCOL11, AtCOL13*, and *AtCOL14*, indicating that gene loss occurred in the process of evolution. This results are basically consistent with the previous phylogenetic tree analysis. As mentioned earlier, there should be six homologous copies of each *Arabidopsis* gene in *B. napus* genome. As 17 *COL* genes were identified in *Arabidopsis* (Robson et al., 2001). In theory, there should be 102 *COL* genes in *B. napus* after whole genome replication, but only 33 *BnaCOL* genes were identified in this study, indicating that about 67.7% of them were lost after whole genome replication. We hypothesized that during the evolution of *B. napus*, the *COL* genes may have undergone strict purification and selection, which play a key role in the maintenance of gene number.

### Functional Expression Diversity of *BnaCOL* Genes in *Brassica napus*

We analyzed expressional pattern of 33 *BnaCOL* genes in the different tissues using public data. The results showed that they were expressed in different tissues and their expression patterns in different tissues were different. A 81.8% of *BnaCOL* genes were highly transcribed in leaves and sepals and main expression of five genes were found in pollen and buds (*BnaCOL5, BnaCOL9, BnaCOL10, BnaCOL21*, and *BnaCOL29*). *BnaCOL6, BnaCOL7*, and *BnaCOL23* showed higher transcription levels in siliques while *BnaCOL22* only highly expressed in seeds but lower in other tissues. These results indicate that the *BnaCOL* gene family may play a significant role in all stages of rapeseed growth and development. Since 81.8% of *BnaCOL* genes mainly expressed in leaves, they may have a potential key role in leaves to response to environmental factors like light condition. Previous studies had shown that CO initiated the transcriptional expression of FT, which in turn transferred FT proteins from leaf phloem to shoot tip meristem, thereby activate flowering (Shim et al., 2017). Hence, the high expression of *BnaCOL* genes help to initiate FT transcription, consequently promote flowering.

The study results of *COL* genes in *Arabidopsis* provide reference and clue for the *BnaCOL* genes function. Previous studies had identified that overexpression of *AtCOL1* in *Arabidopsis* shorten the circadian cycle (Ledger et al., 2001). And *AtCOL3, AtCOL4, AtCOL5, AtCOL8*, and *AtCOL9* were all involved in the regulation of flowering under different photoperiod conditions (Cheng and Wang, 2005; Datta et al., 2006; Hassidim et al., 2009; Takase et al., 2012; Steinbach, 2019). Hence, in order to further explore the role of *BnaCOL* genes in the photoperiod pathway, we selected nine *Arabidopsis* homologous genes that regulate flowering time (*BnaCOL3, BnaCOL5, BnaCOL11, BnaCOL12, BnaCOL15, BnaCOL16, BnaCOL23, BnaCOL30*, and *BnaCOL33*) and detected the diurnal expression profiles of these nine genes within 24 h under LD and SD illumination treatments. The RT-qPCR results showed that most members had different daily expression patterns between LD and SD conditions, revealing the functional difference and divergence of the *BnaCOL* family members in *B. napus*.

The genes of the same evolutionary branch between species are homologous or closely related and their biological functions are roughly the same. Notably, previous studies have shown that *AtCOL1* and *AtCOL4* transcription levels are regulated by circadian rhythms (Ledger et al., 2001; Steinbach, 2019). Our results showed that *BnaCOL16*, as a homologous gene of *AtCOL1*, was expressed at exactly the same level as *AtCOL1* in LD and SD. Therefore, it is speculated that *BnaCOL16* has similar functions to *AtCOL1*. Besides, *AtCOL4* is not only a flowering inhibitor under LD and SD (Steinbach, 2019), but also participates in ABA and salt stress responses (Min et al., 2015). Our RT-qPCR results showed that *BnaCOL30*, as a homolog of *AtCOL4*, had a circadian rhythm expression profile consistent with that of *AtCOL4*. And the response to abiotic stimuli was also detected in the GO annotation of *BnaCOL30* gene. This implied that *BnaCOL30* may have the same function as *AtCOL4*. In the course of evolution, structurally similar genes may sometimes diverge functionally within or between species. Previous studies have shown that overexpression of *AtCOL5* makes plants early flowering in SD light conditions (Hassidim et al., 2009). In our study, the expression levels of *AtCOL5* and its homologous gene *BnaCOL15* were completely opposite under SD light conditions. The *BnaCOL15* gene may have functional differentiation and its specific function needs to be verified by subsequent experiments. In our results, the expression level of *BnaCOL11* is regulated by the circadian rhythm. But the *AtCOL7* gene, which is closely related to *BnaCOL11*, has only reported the function of regulating the branching and shading response of *Arabidopsis thaliana* (Wang et al., 2013), as well as linking light perception with auxin (Zhang et al., 2014). In addition, the *BnaCOL* genes exhibit several types of circadian rhythms, suggesting that functional differences in the *BnaCOL* family responsive to multiple aspects of plant development, including the regulation of flowering. Although the *COL* genes are highly conserved among species, it will also undergo functional differentiation in the course of evolution to adapt to environmental changes. The analysis of *cis*-acting elements in the upstream sequence of *BnaCOL* promoters revealed that *BnaCOL* genes contained elements of light response, hormone response, growth regulation and abiotic-stress response. However, only the role of the *BnaCOL* family in the photoperiod pathway was identified in this study, there are many additional unknown functions of the *BnaCOL* gene family required to be explored.

## Conclusion

In summary, a total of 33 *BnaCOL* genes were identified in *B. napus* and these genes were distributed unevenly on 16 chromosomes. The phylogeny, gene structure, conserved motifs and three-dimensional structure of the COL proteins were analyzed. These genes were classified into three subfamilies and relatively conservative gene structures and motifs were found in the same subfamily. In addition, the *BnaCOLs* promoter region have light-responsive *cis*-elements, as well as a variety of *cis*-acting elements related to hormones and abiotic-stress response. Subsequently, GO annotation and enrichment analysis of *BnaCOL* genes lead us to conclude that most genes are annotated in light signal response and transmission, photoperiod response, flowering regulation, circadian rhythm, etc. The collinearity analysis found 37 pairs of large-segment repetitive genes in *B. napus.* Based on comparative genomics research, the *COL* genes of *B. napus* had undergone polyploidization and different degrees of loss and expansion. We also analyzed the expression patterns of the *BnaCOL* genes in different tissues of rapeseed, which indicated that the *BnaCOL* gene family were of great importance at various developmental stages in *B. napus.* Besides, we tested the diurnal expression profiles of 9 *BnaCOL* genes under LD and SD conditions. Most members showed different daily expression patterns between LD and SD conditions, revealing the functional differences of the *BnaCOL* family in *B. napus*. In general, this article comprehensively analyzed the conservation and divergence of *BnaCOL* family genes functions, which provided a biological basis for the further functional identification of *COL* genes in cruciferous plants.

## Data Availability Statement

The datasets presented in this study can be found in online repositories. The names of the repository/repositories and accession number(s) can be found in the article/Supplementary Material.

## Author Contributions

YC, WW, and JL designed the experiments. YC and RZ performed the experiments. YC, QH, and JL analyzed the data. YC wrote the manuscript. All authors reviewed and approved the manuscript.

## Conflict of Interest

The authors declare that the research was conducted in the absence of any commercial or financial relationships that could be construed as a potential conflict of interest.

## Publisher’s Note

All claims expressed in this article are solely those of the authors and do not necessarily represent those of their affiliated organizations, or those of the publisher, the editors and the reviewers. Any product that may be evaluated in this article, or claim that may be made by its manufacturer, is not guaranteed or endorsed by the publisher.

## Footnotes

1
http://cbi.hzau.edu.cn/bnapus/

2
http://pfam.xfam.org/

3
http://smart.embl.de/

4
https://www.ncbi.nlm.nih.gov/cdd/

5
http://web.expasy.org/protparam/

6
https://www.genscript.com/wolf-psort.html

7
http://gsds.gao-lab.org/

8
http://www.sbg.bio.ic.ac.uk/phyre2/html/page.cgi?id=index

9
http://bioinformatics.psb.ugent.be/webtools/plantcare/html

10
http://eggnog-mapper.embl.de/

11
http://yanglab.hzau.edu.cn

12
http://www.heatmapper.ca

13
https://biodb.swu.edu.cn/qprimerdb/

## Supplementary Material

Supplementary Figure 1 Multiple sequence alignment of AtCOL and BnaCOL proteins.

Supplementary Figure 2 Three-dimensional structure prediction of B-box domain of BnaCOL proteins. **(A)** Two B-box domains from group I. **(B)** A B-box domain and a second divergent B-box domain from group II. **(C)** One B-box domain from group III. The images are from dark blue to dark red, indicating from the N end to the C end.

Supplementary Figure 3 GO annotation of *BnaCOL* genes in *B. napus*.
