# Supplementary figures and images for: Conservation and Divergence of the CONSTANS-Like (COL) Genes Related to Flowering and Circadian Rhythm in Brassica napus
Source: Front Plant Sci. 2021 Nov 22;12:760379. doi: 10.3389/fpls.2021.760379 (PMC8645894; doi:10.3389/fpls.2021.760379)

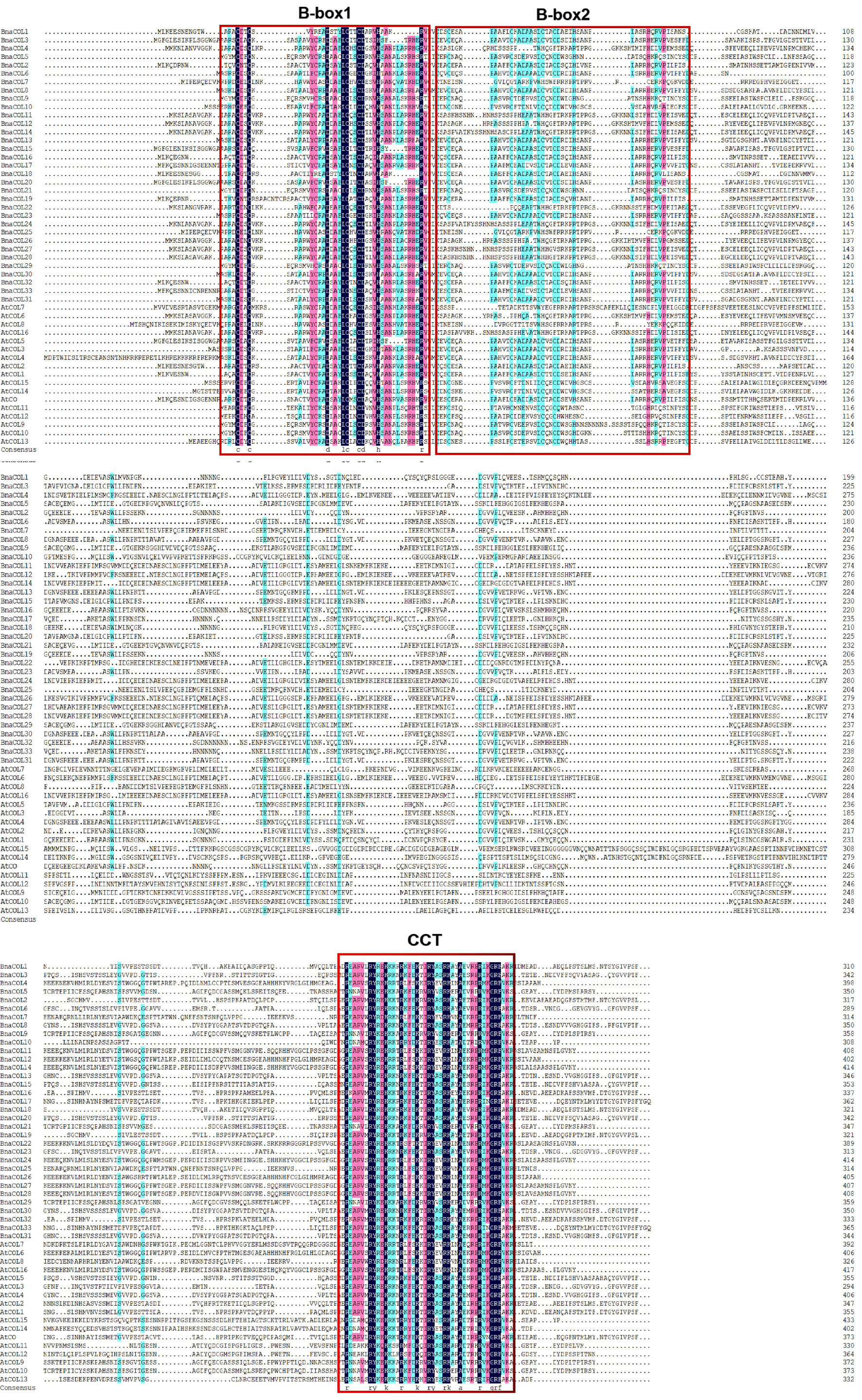

Supplement: Supplementary Figure 1 — Multiple sequence alignment of AtCOL and BnaCOL proteins. [file Image_1.JPEG]

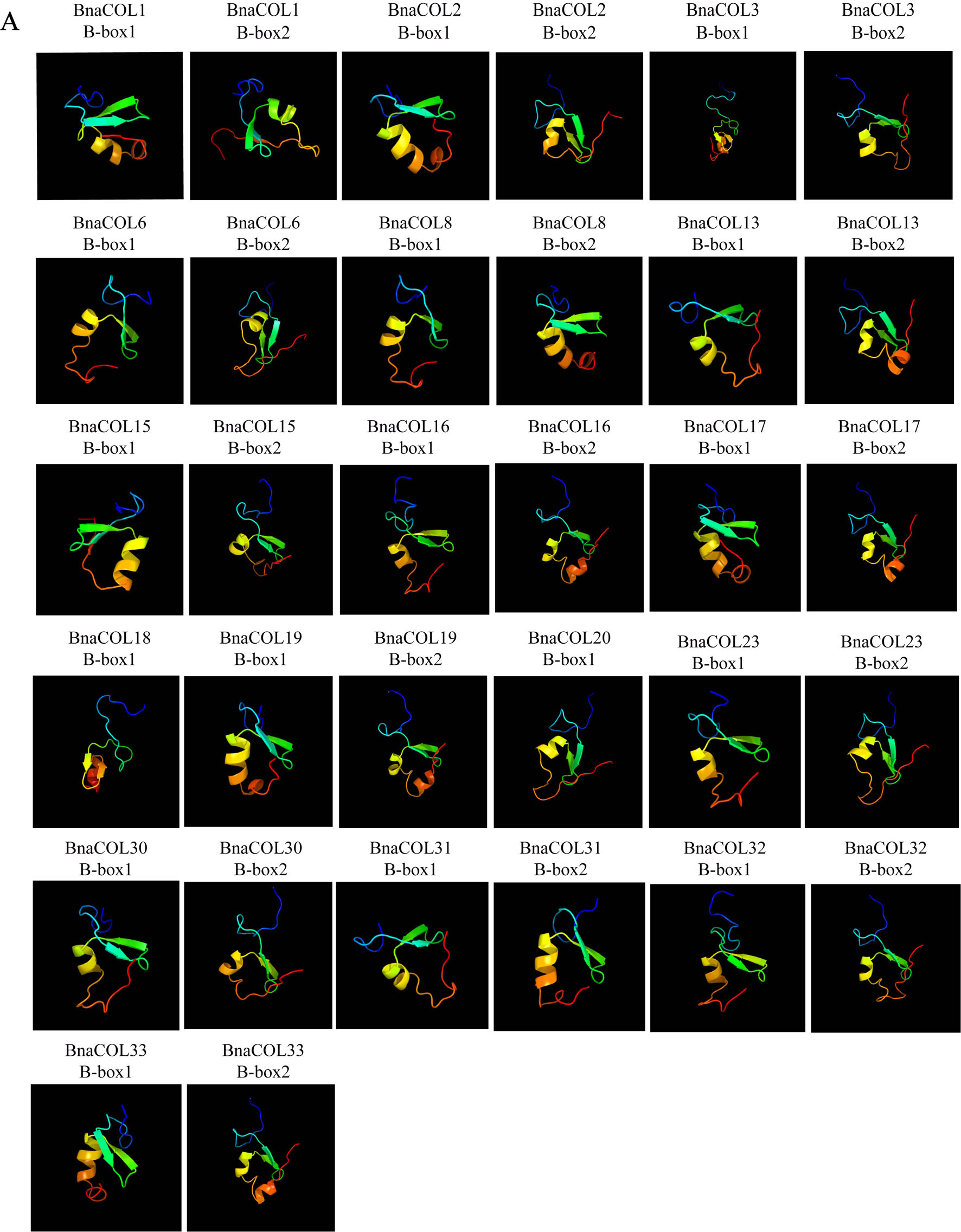

Supplement: Supplementary Figure 2 — Three-dimensional structure prediction of B-box domain of BnaCOL proteins. (A) Two B-box domains from group I. (B) A B-box domain and a second divergent B-box domain from group II. (C) One B-box domain from group III. The images are from dark blue to dark red, indicating from the N end to the C end. [file Image_2.JPEG]

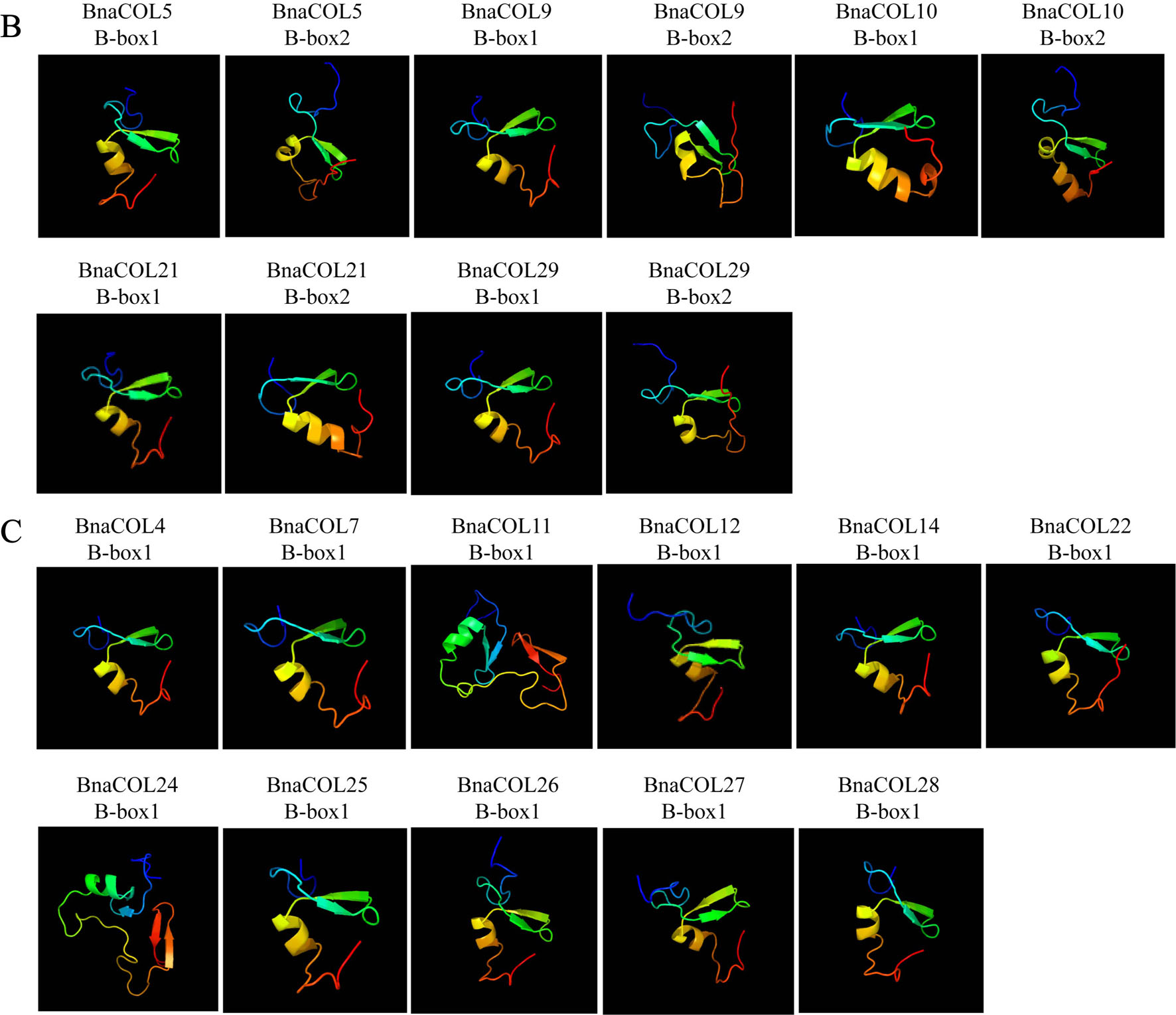

Supplement: Supplementary Figure 3 — GO annotation of BnaCOL genes in B. napus. [file Image_3.JPEG]

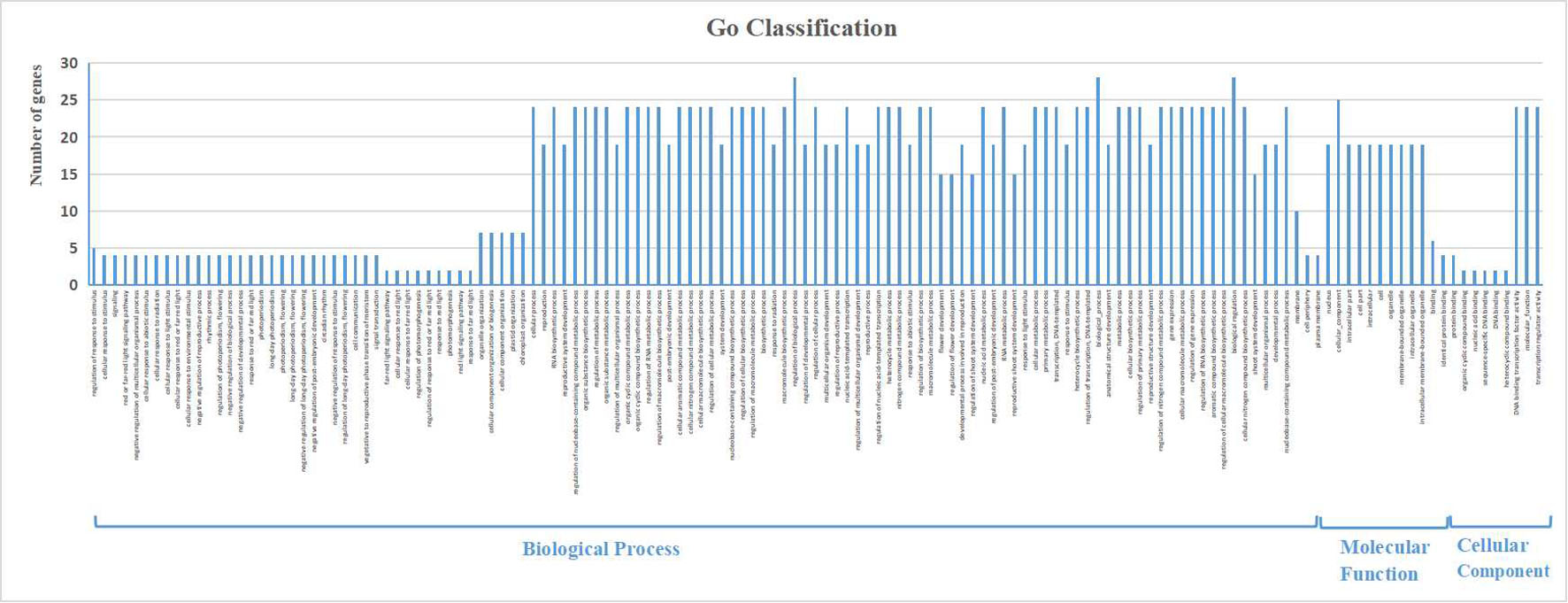

Supplement: Supplementary file 4 [file Image_4.JPEG]
